# Enhancing Educational and Clinical Trainer's Support and Experience: A Quality Improvement Initiative

**DOI:** 10.1192/bjo.2024.316

**Published:** 2024-08-01

**Authors:** Ashma Mohamed, Zafrina Majid, Abiola Ige, Katie Kopala, Mary-Anne Cotton

**Affiliations:** Surrey and Borders Partnership NHS Foundation Trust, Surrey, United Kingdom

## Abstract

**Aims:**

The GMC Trainer's survey 2022 identified nearly two in ten (18%) trainers do not agree that their employer provides a supportive environment for everyone regardless of background, beliefs, or identity. A striking 52% of doctors working as trainers are identified as being at moderate to high risk of burnout. Surrey and Borders Partnership NHS Foundation Trust(SABP) has 63 active educational and clinical trainers.

We aim to enhance the overall experience of Educational and Clinical Trainers in SABP by gaining insights into their views and experiences and identifying key areas for improvement to support trainers in their roles, thereby contributing to a more resilient healthcare workforce.

**Methods:**

We devised a 16 item questionnaire to gather anonymous data on trainers' experiences and views in their roles. Our study utilised an observational quantitative and qualitative cross-sectional design. Data capture was done on Microsoft Forms and analysed using Excel.

**Results:**

We had 70% response rate, 90% agreed or strongly agreed they had adequate support and training, 95% feel able to fulfil educational CPD for appraisal however only 83% were able to complete reflections on trainee feedback. 93% agreed or strongly agreed that they enjoy being a trainer but only 67% agreed or strongly agreed that they knew how to access support if they felt burnt out. Only 43% felt that they had adequate time in their schedule to provide supervision. Analysis of responses stratifying International medical graduates and years of experience being a trainer did not identify additional needs.

**Conclusion:**

Effective trainers are fundamental in shaping future doctors. Our survey results highlighted that a high percentage of trainers enjoy their role. Based on the results, strategies were identified to improve support that can be implemented through trainers’ drop-in sessions, advertising trainers' training sessions with more notice and developing the resources on the intranet including improving content and adding videos of training sessions. We also identified that appraisal and revalidation requirements for trainers, trainee surveys needed to be better advertised to improve feedback rates. We recommended that a document on the online appraisal platform (SARD) be added to clarify the requirements for appraisal and revalidation, and how these can be met. We suggested that Associate Medical Directors consider the need to ringfence time for educational and clinical trainers in their job plans.

